# Phytochemical and functional characterization of fermented Yerba mate using *Rhizopus oligosporus*

**DOI:** 10.1186/s13568-023-01600-4

**Published:** 2023-09-09

**Authors:** So-Hyung Kwak, Hayeong Kim, Ji hyeon Jeon, Kunal Pal, Dong-Hyun Kang, Doman Kim

**Affiliations:** 1 Department of Agricultural Biotechnology, College of Agriculture and Life Sciences, Seoul National University, Seoul, 08826 Republic of Korea; 2 The Institute of Food Industrialization, Institutes of Green Bioscience & Technology, Seoul National University, Gangwon-do, 25354 Republic of Korea; 3 Graduate School of International Agricultural Technology, Center for Food and Bioconversionce, Seoul National University, Gangwon-do, 25354 Republic of Korea; 4 Department of Biotechnology and Medical Engineering, National Institute of Technology, Rourkela, 769008 India; 5 Department of Food and Animal Biotechnology, Department of Agricultural Biotechnology, Center for Food and Bioconvergence, and Research Institute for Agricultural and Life Sciences, Seoul National University, Seoul, 08826 Republic of Korea; 6 Fervere Campus Corporation, Gangwon-do, 25354 Republic of Korea

**Keywords:** Solid fermentation, Yerba mate, *Rhizopus oligosporus*, α-glucosidase, Antioxidant

## Abstract

Solid-state fermentation (SSF) was used to enhance the bioactive compounds and biological properties of food materials, such as buckwheat, turmeric, and ginseng. This study was investigated the effects of SSF for up to 10 days using *Rhizopus oligosporus* on Yerba mate (*Ilex paraguariensis* St. Hilaire). The total phenolic content of Yerba mate rose to 20% after 1 day fermentation. The saponin contents of Yerba mate rose to 38% after 7 day fermentation. Furthermore, chlorogenic acid, caffeic acid, and caffeine levels were increased up to 27.74% by fermentation, as determined by UPLC-MS analysis. ORAC and FRAP assays showed that the antioxidant activities of Yerba mate were enhanced 1.9- and 1.14-fold after 1 day fermentation. In addition, its inhibitory activities against yeast α-glucosidase and nitric oxide release in LPS-stimulated RAW264.7 cells were higher than in the unfermented Yerba mate. Moreover, taste sensory analysis using an electronic tongue sensory system showed that the flavor of Yerba mate after 1 day fermentation was similar to that of the unfermented Yerba mate. These results suggested that solid fermentation using *R. oligosporus* is conducive to producing Yerba mate with enhanced biological properties.

**Supplementary Information:**

The online version contains supplementary material available at 10.1186/s13568-023-01600-4.

## Introduction

Solid-state fermentation (SSF) has attracted increasing interest as a bioprocess for applications for nutritional enrichment, and the production of bioactive products, including organic acids, aroma compounds, enzymes, and biofuels (Pandey et al. 2000). In SSF, microorganisms can grow on solid material as moist nutrition in an absorbed state (Krishna 2005). The advantages of SSF are high volumetric productivity, increased enzyme yield, methodological simplicity, and low risk of bacterial contamination due to low moisture content compared to submerged liquid fermentation (Ikasari and Mitchell 1994). *Rhizopus oligosporus* used in Tempeh, a traditional Indonesian food, is a GRAS (generally regarded as safe) organism that produces various extracellular enzymes, including cellulases, hemicellulases, xylanases, amylases, chitinases, proteases, lipases, and phosphatases (Huang et al. 2019; Miszkiewicz et al. 2003; Nguyen Thai et al. 2014). Previous studies have reported that SSF using *R. oligosporus* enhances the bioactive compounds and functional properties of buckwheat, turmeric, ginseng, quinoa, and soybean (Hur et al. 2018; Lee et al. 2020; Lim et al. 2023, 2022; McCue and Shetty 2003; Park et al. 2017).

*Ilex paraguariensis* St. Hilaire (Yerba mate) is growing in South America (Ferrari Pereira Lima et al. 2012) and its leaves are used to produce Yerba mate infusion (Heck and De Mejia 2007). Concentrated extracts and drinks of Yerba mate have rapidly become popular around the world and are used in the pharmaceutical, food, and cosmeceutical industries owing to their bioactive compounds with pharmacological effects against chronic diseases (Gawron-Gzella et al. 2021). Yerba mate leaves are dried and roasted to produce tea. Yerba mate tea is dried very slowly which is different compared to green tea that dried mainly via a fast and high-temperature process (Heck and De Mejia 2007). It contains 80.71% carbohydrates, 4.09% protein, 0.9% fats, minerals, and vitamins (Berté et al. 2011; Heck and De Mejia 2007). In addition, it also contains bioactive compounds, such as purine alkaloids, phenolic acids, flavonoids, and terpenes (saponins), which contribute to its biological properties, including antiobesity, antioxidant, antidiabetic, anti-inflammatory, anticancer, and antimicrobial effects (Arçari et al. 2009; Berté et al. 2011; Filip et al. 2001; Heck and De Mejia 2007). Our previous study showed that the contents of ginsenosides, the main bioactive compounds of wild-simulated ginseng leaves, were increased by up to 343% by fermentation using *R. oligosporus*. The antioxidant properties determined by ferric-reducing antioxidant power (FRAP) and oxygen radical absorbance capacity (ORAC) of wild-simulated ginseng leaves were enhanced by up to 63% (Lim et al. 2023). Another study showed that wild turmeric had increased levels of total phenolic compounds, total flavonoid-curcuminoid, curcumin, demethoxycurcumin, and bisdemethoxycurcumin by fermentation using *R. oligosporus* (Lim et al. 2022). Moreover, fermented wild turmeric showed a 47% increase in ORAC, 125% increase in FRAP, 44% increase in the inhibitory effect against nitric oxide (NO) formation in lipopolysaccharide (LPS)-stimulated RAW 264.7 cells, and 52% increase in melanin formation by B16F10 mouse melanoma cells (Lim et al. 2022). This study aims to enhance the bioactive compounds of Yerba mate via solid-state fermentation using *R. oligosporus*. The total polyphenol contents, total saponin contents, ferulic acid, chlorogenic acid, caffeic acid, rutin, and caffeine in fermented Yerba mate were determined. The antioxidant properties by ferric-reducing antioxidant powder (FRAP) and oxygen radical absorbance capacity (ORAC) assays and anti-inflammatory activity in LPS-stimulated RAW264.7 cells of fermented Yerba mate were analyzed. Furthermore, the effects of fermentation on Yerba mate sensory taste were evaluated by an electronic tongue sensor system. The results confirmed the utility of fermented Yerba mate as a functional material in the pharmaceutical, food, and beverage industries to promote health.

## Materials and methods

### Materials and microbial strain

*Rhizopus oligosporus* (*R. oligosporus*) KCCM 11948P was obtained from Korean Culture Center of Microorganisms (KCCM, Seoul, Korea) (Park et al. 2017). RAW264.7 murine macrophage cells were obtained from the Korean Cell Line Bank (KCLB, Seoul, Korea). Yerba mate was purchased from NATURE (Chungju-si, Korea). Ferulic acid, gallic acid (GA), chlorogenic acid, caffeic acid, 6-hydroxy-2,5,7,8-tetramethylchromane-2-carboxylic acid (Trolox), Folin-Ciocalteu reagent, lipopolysaccharides (LPS), α-glucosidase from *Saccharomyces cerevisiae*, and dimethyl sulfoxide (DMSO) were bought from Sigma-Aldrich (St. Louis, MO, USA). Caffeine was obtained from Tokyo Chemical Industry Co., Ltd (Tokyo, Japan) and EZ-Cytox was bought from DOGEN BIO (Seoul, Korea). Rutin, and 2,2′-azobis (2-methylpropionmidine) dihydrochloride (AAPH) were purchased from Acros Organics (Geel, Belgium). Fluorescein was bought from Alfa Aesar (Haverhill, MA, USA). Dulbecco’s modified Eagle’s medium (DMEM) and fetal bovine serum (FBS) were purchased from Gene Depot (Barker, TX, USA), penicillin and streptomycin (PS) were obtained from Invitrogen (Carlsbad, CA, USA).

### Fermentation of Yerba mate using *R. oligosporus*

*R. oligosporus* was grown to obtain spores on potato dextrose agar (PDA) for 5 days at 30 °C (Park et al. 2017). Ground and dry Yerba mate leaves (10 g) were mixed with 30 mL of water for 12 h and steamed at 121 °C for 15 min. Then, Yerba mate was inoculated with 4 × 10^5^ spores/g and incubated at 30 °C for 1–10 days. After that, fermented yerba mate was frozen at − 75 °C and then lyophilized at − 45 °C under a pressure of 10 Pa using Eyela FD-550 (Rikakikai, Tokyo, Japan) for further study.

For extraction, 70% ethanol (200 mL) was added to samples (4 g) and sonicated using an ultrasonic homogenizer (Korea Biotech, Seongnam-si, Korea) with a power of under 400 W for 10 min (on for 3 s and off for 4 s) at room temperature. The samples were then sonicated in a water bath sonicator (DAIHAN Scientific, Gangwon-do, Korea) for 30 min at 60 °C. Supernatant was gained by centrifuging at 8000 × *g* for 15 min, and then filtered by paper filter No. 1 (Whatman, Piscataway, NJ, USA). Ethanol in the sample was discard through evaporation (Heidolph Instruments, Schwabach, Germany) at 45 °C, and lyophilized as above. The extraction yield was calculated as follows:

$$\mathrm{Yield} (\%)=\frac{extract\, mass\,\, (\mathrm\,{g})}{Yerba\, mate\, mass (\mathrm\,{g})}\times 100$$

### Phytochemical characterization of fermented Yerba mate

#### Analysis of ergosterol using ultra-performance liquid chromatography-tandem mass spectrometry (UPLC-MS)

Ergosterol in nonfermented and fermented Yerba mate was extracted and analyzed by UPLC-MS with a PDA detector using a BEH C_18_ column (2.1 × 100 mm, 1.7 μm) as described previously (Lim et al. 2022). Ergosterol ranging from 0.1 to 60.0 μg/mL was utilized as standard (Additional file 1: Table S1).

#### Total phenolic content (TPC)

TPC of samples was analyzed using the Folin-Ciocalteu method with GA as the standard (Kim et al. 2003). 15 μL of Folin-Ciocalteu’s phenol reagent and 15 μL of Na_2_CO_3_ (10%, w/v) were added to a 96-well plate containing 120 μL of the sample. The plate was kept for 30 min in the dark, then measured at 760 nm using a SpectraMax M3 microplate reader (Molecular Devices, San Jose, CA, USA). TPC was shown as mg gallic acid equivalent (GAE)/g dry weight (g DW).

#### Total saponin content (TSC)

TSC of unfermented and fermented Yerba mate was analyzed using vanillin-sulfuric acid assay (V. Le et al. 2018). Briefly, 8% (w/v) vanillin in ethanol (100 μL) and 72% (v/v) sulfuric acid (1 mL) were added to the plate containing sample or oleanolic acid (100 μL), kept at 60 °C for 10 min in the water bath, and then cooled on ice for 5 min. After that, the plate was measured at 544 nm using a M3 spectrophotometer. The TSC is displayed as mg oleanolic acid equivalent (OAE) per gram of Yerba mate (mg OAE/g DW).

#### Analysis of bioactive compounds using UPLC-MS

UPLC-MS was conducted to determine caffeic acid, ferulic acid, chlorogenic acid, caffeine, and rutin contents in unfermented and fermented Yerba mate as described previously (Lim et al. 2022). Electrospray ionization (ESI) was performed in positive ion mode with single ion recording (SIR) (*m*/*z* 355.0 for chlorogenic acid, *m*/*z* 181.10 for caffeic acid, *m*/*z* 195.13 for ferulic acid, and *m*/*z* 195.17 for caffeine). Negative ESI was controlled with SIR (*m*/*z* 609 for rutin). The capillary energy was 1.3 kV. The cone voltages used to assess bioactive compounds were 5 V for chlorogenic acid, 10 V for ferulic acid, caffeic acid, and caffeine, and 20 V for rutin. A calibration curve using the external standard method was prepared with chlorogenic acid (0.1–10.0 μg/mL) and other compounds (0.1–5.0 μg/mL) (Additional file 1: Table S2).

### Antioxidant properties of fermented Yerba mate

The antioxidant activities of unfermented and fermented Yerba mate were analyzed based on ORAC with Trolox as a standard and FRAP, as described previously with slight modifications (Benzie and Strain 1996; Mok et al. 2020). For the ORAC assay, 90 μL of 25 nM fluorescein, a 10 μL sample, and 25 mM AAPH (100 μL) for radical induction were added to a 96-well plate and monitored every 3 min at 485 nm excitation wavelength and at 538 nm emission wavelength using a SpectraMax M3 microplate reader for 2 h 37 °C. The net area under the curve (AUC) was obtained by subtracting the AUC of the blank from the AUC of the tested sample. The antioxidant activity was displayed as μmol Trolox equivalent per gram of sample (µmol TE/g DW).

Unfermented or fermented Yerba mate sample or ferrous sulfate heptahydrate (200–2000 μM) (6 μL) were mixed with FRAP working solution (180 μL) comprising of 20 mM ferric chloride anhydrous solution, 10 mM 2,4,6-tripyridyl-s-triazine, and 0.3 M sodium acetate buffer (pH 3.6) with ratio of 1:1:10 (v/v/v) and water (18 μL) for 30 min. Then the reaction was recorded at 593 nm using a SpectraMax M3 microplate reader. FRAP was displayed in μmol of Fe^2+^ per gram of sample (μmol Fe^2+^/g DW).

### α-Glucosidase inhibition assay

The inhibition effects of unfermented and fermented Yerba mate on α-glucosidase activity were analyzed following the procedure described by Ortíz-Martinez et al. (2016) using pNPG (*p*-nitrophenyl-α-d-glucopyranoside) as the substrate with some modifications. Different concentrations of the sample (0.375–2.0 mg/mL) were added in the reaction mixture containing 0.1 U/mL α-glucosidase in 50 mM potassium phosphate buffer (pH 6.8), kept at 37 °C for 5 min, and started by adding 500 μM pNPG for 10 min at 37 °C. Next, 250 mM Na_2_CO_3_ was mixed with the reaction mixture with a ratio of 1:1 (v/v). The *p*-nitrophenol formation was determined at 405 nm using a SpectraMax M3 microplate reader. The positive and negative control was epigallocatechin gallate (EGCG) and DMSO. The inhibitory activity was calculated relative to the control.

### Cell viability assay

RAW264.7 macrophage cells after seeding on 96-well plates at 2 × 10^4^ cells/well in DMEM comprising of 10% (v/v) FBS, 100 U/mL penicillin, and 100 μg/mL streptomycin for 24 h were treated with sample at 12.5–400 μg/mL. Then, the plate was incubated at 37 °C in 5% CO_2_. RAW264.7 cells without treated sample were used as controls. After 24 h, the culture medium was discarded and solution comprising of Ez-CyTox solution (100 μL) and DMEM with the ratio of 1:9 was added to the plate. Then, the plate was incubated at 37 °C for 1 h, then read at 450 nm using a SpectraMax M3 microplate reader. The cell viability was estimated relative to the control.

### NO production

RAW264.7 macrophage cells after seeding on 96-well plates at 2 × 10^4^ cells/well were treated with sample (50 μg/mL) and LPS (1 μg/mL), followed by incubation at 37 °C in 5% CO_2_. Cells treated with 100 μM indomethacin and LPS (1 μg/mL) were used as controls. After 24 h, culture supernatant (80 μL) was mixed with Griess reagent (80 μL), kept at 22 °C for 20 min, and read at 540 nm using a SpectraMax M3 microplate reader (Hur et al. 2018).

### Taste sensory evaluation using an electronic tongue sensor system

Taste sensory evaluation of unfermented, 1-, 3-, 5-, 7-, and 10 day fermented Yerba mate was performed using a TS-5000Z electronic tongue sensor system (Insent Inc., Atsugi-Shi, Japan). Umami (AAE), astringency (AE1), sourness (CA0), bitterness (C00), and saltiness (CT0) sensors were utilized to dissect the taste of unfermented and fermented Yerba mate (Kobayashi et al. 2010). Samples of 3 g of raw materials were solubilized in 120 mL of water. Taste value was determined by converting sensor output using the SA402B taste analysis application.

### Statistical analysis

All data are shown as the mean ± standard deviation (SD) by triplicate experiments. One-way analysis of variance (ANOVA) followed by Tukey’s multiple comparison test using SPSS for Windows software (version 23.0; SPSS Inc., Chicago, IL, USA) was used for the analysis of differences among groups (*p* < 0.05). Differences in the α-glucosidase inhibition assay were analyzed using one-way ANOVA following the Duncan method.

## Results

### Solid-state fermentation of Yerba mate using *R. oligosporus*

**Fig. 1:**
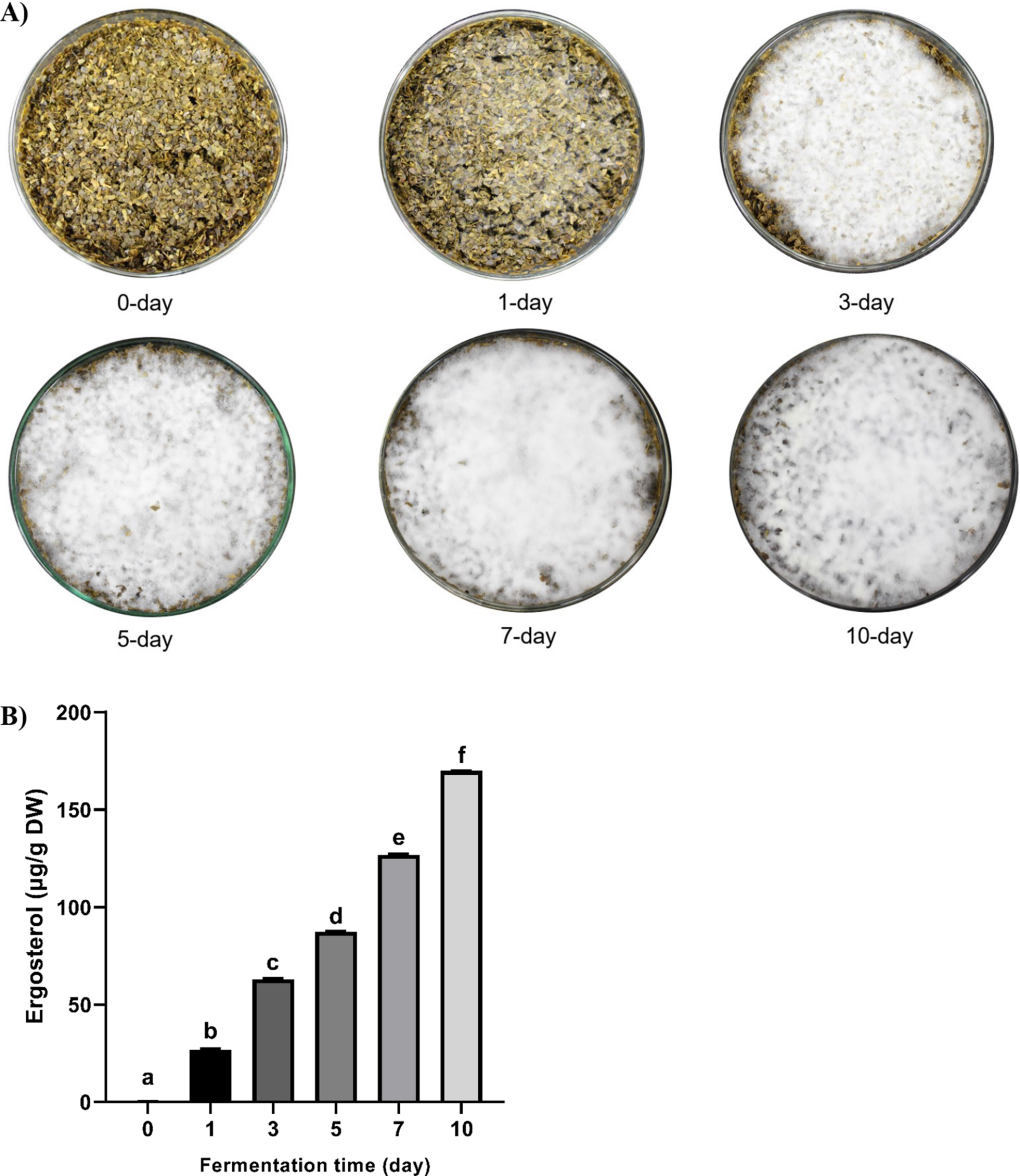
The growth of *R. oligosporus* (**A**) and ergosterol content (**B**) in Yerba mate during fermentation using *R. oligosporus*. Data are means ± standard deviation (SD) of three independent experiments. Different letters indicate statistical differences by Tukey’s multiple comparison test at p < 0.05

The growth of *R. oligosporus* on Yerba mate is presented in Fig. 1A. In this study, the growth pattern of *R. oligosporus* on Yerba mate was analyzed using ergosterol. Ergosterol (5,7-diene oxystorol), a key sterol in the membrane of most fungi, has been used to determine the growth of *R. oligosporus* during solid-state fermentation (Douglas and Konopka 2014; Lim et al. 2023). The ergosterol in nonfermented and fermented Yerba mate was shown in Fig. 1B. Ergosterol content rose from 0.37 ± 0.02 μg/g in nonfermented Yerba mate to 169.89 ± 0.14 μg/g after 10 days of fermentation (Fig. 1B). The ergosterol content in Yerba mate was increased with increasing the fermentation time.

### Extraction yield

The extraction yields of nonfermented and fermented Yerba mate at 1 day (1F), 3 day (3F), 5 day (5F), 7 day (7F), and 10 day (10F) were 41%, 41% ± 1%, 43% ± 2%, 44% ± 1%, 45% ± 1%, 45% ± 1%, and 41% ± 1%, respectively.

### Phytochemical characterization of Yerba mate fermented using* R. oligosporus*

#### Total phenolic content

**Fig. 2:**
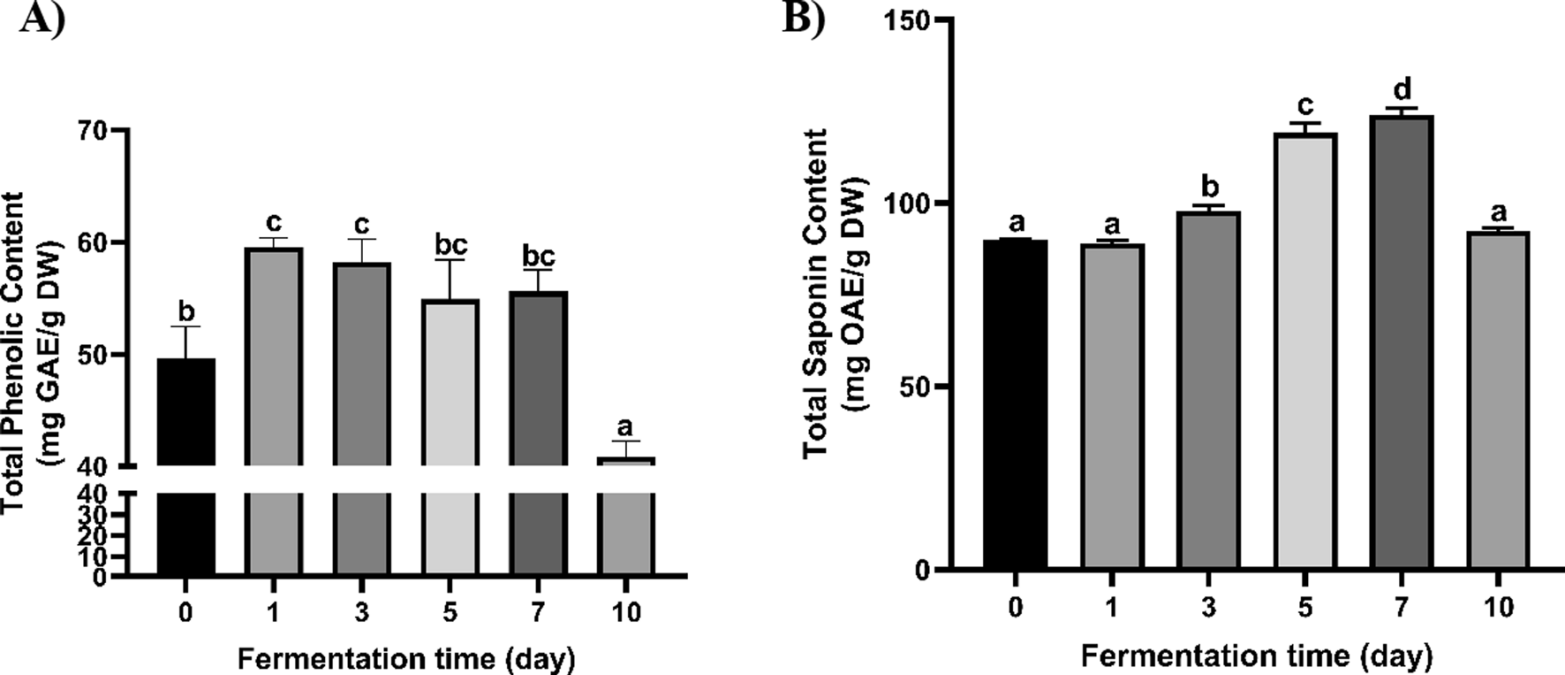
Total phenolic content (**A**) and total saponin content (**B**) of fermented Yerba mate using *R. oligosporus*. Data are means ± standard deviation (SD) of three independent experiments. Different letters indicate statistical differences by Tukey’s multiple comparison test at p < 0.05

TPC of unfermented and *R. oligosporus*-fermented Yerba mate from 1 to 10F is shown in Fig. 2A. The TPC of fermented Yerba mate increased at 1F, then decreased until 7F, and abruptly at 10F. The highest TPC content was obtained after 1 day fermentation. In comparison to TPC of nonfermented Yerba mate, the TPC of 1F yerba mate was increased by 20%, in contrast, TPC of 10F Yerba mate was decreased by 22%.

#### Total saponin contents (TSC)

The TSC in unfermented and *R. oligosporus*-fermented Yerba mate is presented in Fig. 2B. No significant difference in TSC among unfermented and 1F Yerba mate was found (Fig. 2B). However, TSC was significantly increased in 3F, 5F, and 7F Yerba mate (Fig. 2B). The highest TSC of Yerba mate was increased up to 38% after 7 day fermentation.

#### Analysis of bioactive compounds

The concentrations of ferulic acid, chlorogenic acid, caffeic acid, rutin, and caffeine, which are major functional compounds found in Yerba mate that come up with antioxidant, antiobesity, and antimutagenic effects (Kaezer et al. 2012), are expressed in Table 1. Using UPLC-MS to analyze bioactive compounds in Yerba mate, we found that the chlorogenic acid, caffeic acid, and caffeine in 5F Yerba mate increased by 7%, 28%, and 15%, respectively, compared to non-fermented Yerba mate, while its ferulic acid and rutin levels remained unchanged until 5F. In contrast, chlorogenic acid, caffeic acid, and rutin in 10F Yerba mate were reduced by 88%, 744%, and 103%, respectively, while ferulic acid and caffeine increased by 24% and 28%.

**Table 1:** Bioactive compounds of fermented Yerba mate using *R. oligosporus*

Fermented Time (day)	Chlorogenic acid	Caffeic acid	Ferulic acid	Caffeine	Rutin
	μg/mg DW				
0	22.78 ± 0.15^bc^	0.76 ± 0.02^bc^	10.93 ± 0.44^a^	10.67 ± 0.73^a^	6.46 ± 0.21^c^
1	23.59 ± 1.57^bc^	0.86 ± 0.04^c^	11.58 ± 0.24^a^	11.55 ± 0.22^ab^	6.70 ± 0.17^c^
3	23.94 ± 0.76^c^	0.88 ± 0.04^cd^	11.94 ± 0.20^a^	11.99 ± 0.17^b^	6.72 ± 0.08^c^
5	24.29 ± 0.69^c^	0.98 ± 0.04^d^	11.90 ± 0.66^a^	12.29 ± 0.07^b^	6.84 ± 0.17^c^
7	21.14 ± 1.00^b^	0.69 ± 0.02^b^	11.90 ± 0.38^a^	11.89 ± 0.38^b^	5.52 ± 0.19^b^
10	12.25 ± 0.58^a^	0.09 ± 0.07^a^	13.58 ± 0.24^b^	13.63 ± 0.27^c^	3.19 ± 0.05^a^

The data were shown as the mean ± SD. The different letters of the alphabet in each column represent significant differences (*p* < 0.05)

### Antioxidant activity of fermented Yerba mate

**Fig. 3:**
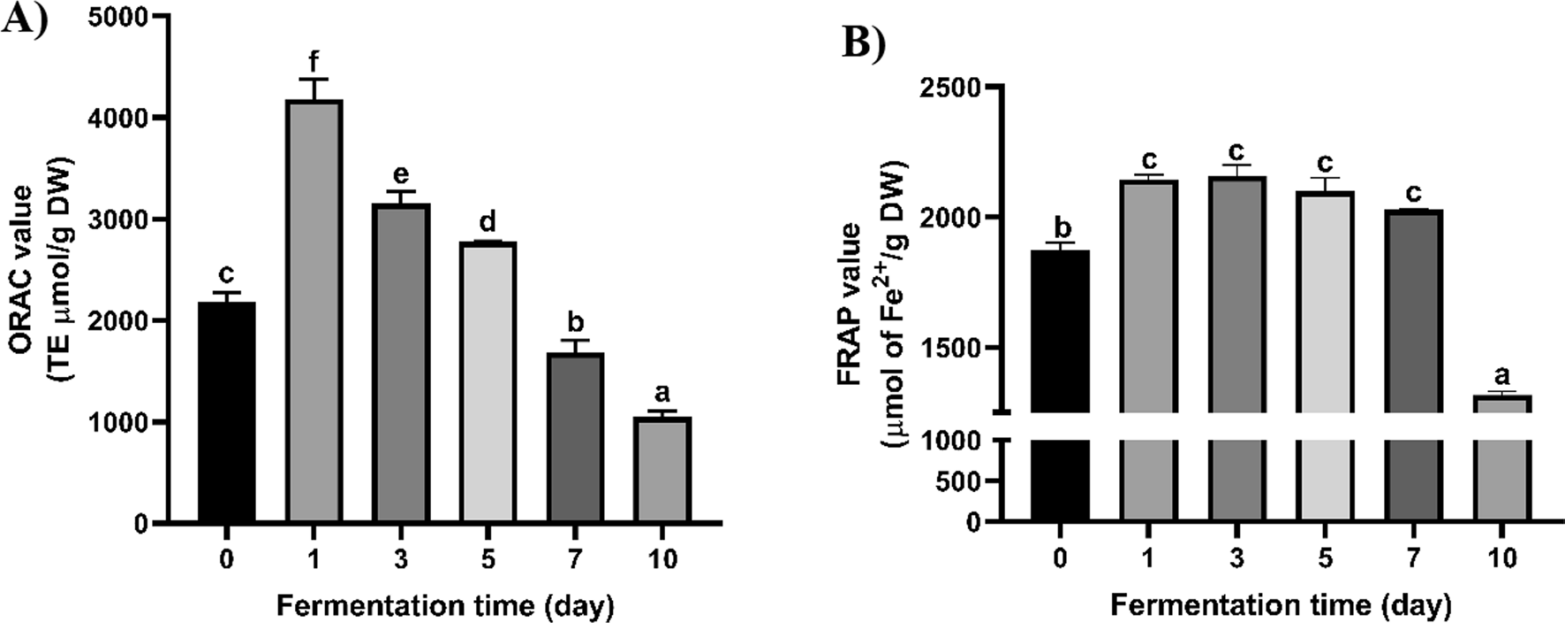
Oxygen radical absorbance capacity (ORAC) (**A**) and ferric reducing antioxidant power (FRAP) (**B**) of fermented Yerba mate using *R. oligosporus*. Data are means ± standard deviation (SD) of three independent experiments. Different letters indicate statistical differences by Tukey’s multiple comparison test at p < 0.05

The antioxidant activities of unfermented and *R. oligosporus*-fermented Yerba mate determined by ORAC and FRAP assays are shown in Fig. 3A, B. The ORAC value of 1F, 3F, and 5F Yerba mate was increased by 92%, 45%, and 27% compared to nonfermented Yerba mate. In contrast, the ORAC value in 7F and 10F Yerba mate was decreased to 30% and 109% compared to the nonfermented Yerba mate.

In comparison to the nonfermented Yerba mate, the FRAP value of 1F, 3F, 5F, and 7F Yerba mate was increased by 15%, 15%, 12%, and 8%, respectively, while the FRAP value of 10F Yerba mate was decreased 42%.

### The inhibitory effects of fermented Yerba mate on α-glucosidase activity

The inhibition of fermented Yerba mate against yeast α-glucosidase is presented in Table 2. The greatest inhibition of fermented Yerba mate against α-glucosidase was obtained after 1F with a 50% inhibitory concentration (IC_50_) of 846 ± 12 μg/mL (Table 2). The inhibitory activity against α-glucosidase showed no significant differences among unfermented, 3F, and 5F Yerba mate. EGCG used as a positive control had an IC_50_ against α-glucosidase activity of 55 ± 1 μg/mL.

**Table 2:** α-glucosidase inhibitory effect of fermented yerba mate

Fermentation day	IC_50_ (μg/mL)
0	896 ± 17^b^
1	846 ± 12^a^
3	879 ± 24^b^
5	890 ± 22^b^
7	949 ± 19^c^
10	1057 ± 15^d^

The data were expressed as the mean ± SD. The different letters of the alphabet represent significant differences (*p* < 0.05)

### Cell viability and inhibitory effects of fermented yerba mate on NO production

**Fig. 4:**
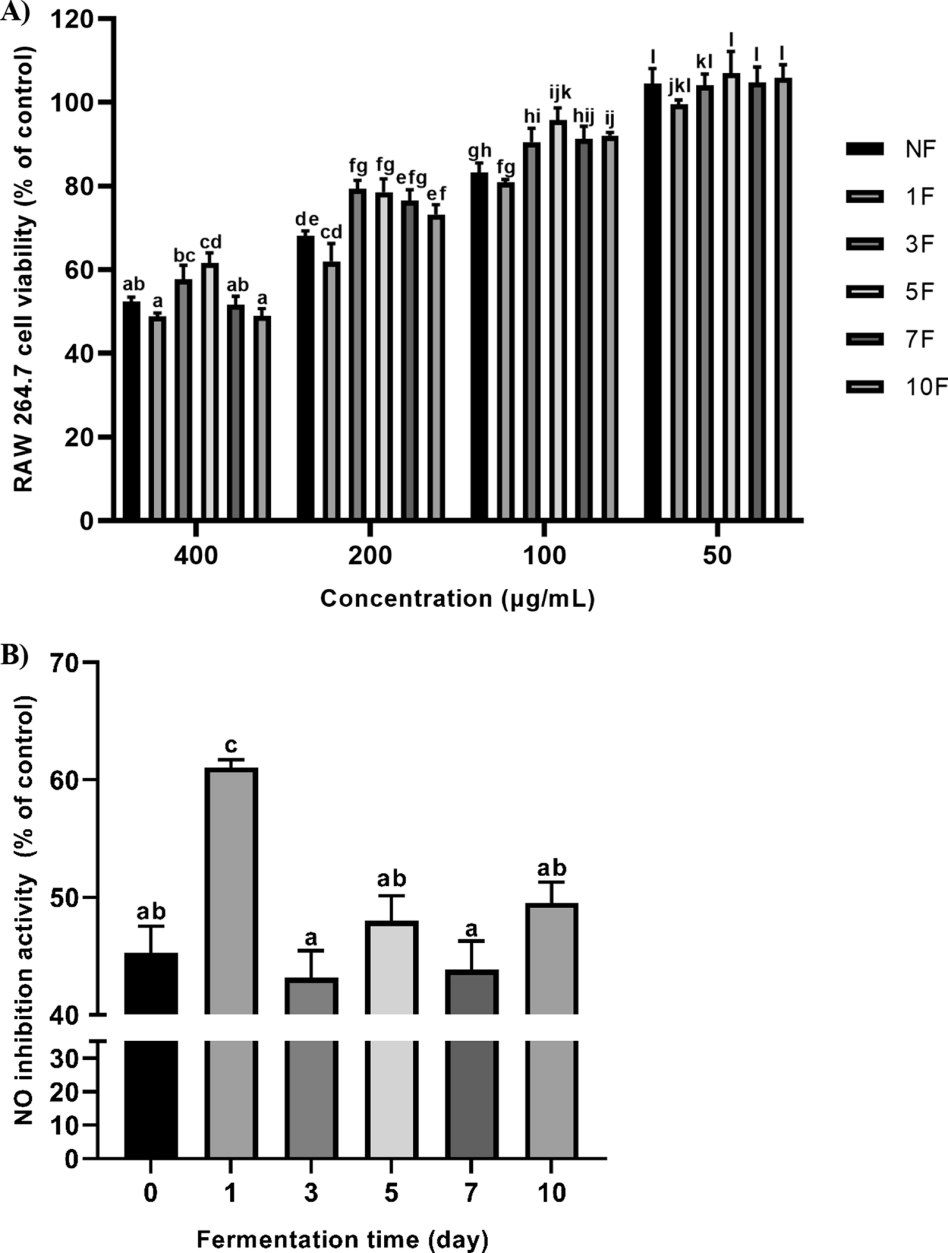
Cell viability (**A**) and inhibitory effects against nitric oxide production in LPS-stimulated RAW264.7 cells (**B**) of fermented Yerba mate using *R. oligosporus*. NF: non-fermented yerba mate; 1F, 3F, 5F, 7F, and 10F: 1 day, 3 day, 5 day, 7 day, and 10 day fermentation of yerba mate. Data are means ± standard deviation (SD) of three independent experiments. Different letters indicate statistical differences by Tukey’s multiple comparison test at p < 0.05

The effects of unfermented and fermented Yerba mate on RAW 264.7 cell viability are presented in Fig. 4A. Cell viability was 100% at 50 μg/mL, so the NO assay was investigated at this concentration. The inhibition of unfermented and fermented Yerba mate against nitric oxide production from LPS-stimulated RAW264.7 cells are presented in Fig. 4B. The NO inhibitory activity of unfermented and 1F Yerba mate was 45 ± 2% and 61 ± 1%, respectively. The NO inhibitory activity showed no significant differences among nonfermented, 3F, 5F, 7F, and 10F Yerba mate (Fig. 4B).

### Taste sensory evaluation of fermented Yerba mate

**Fig. 5:**
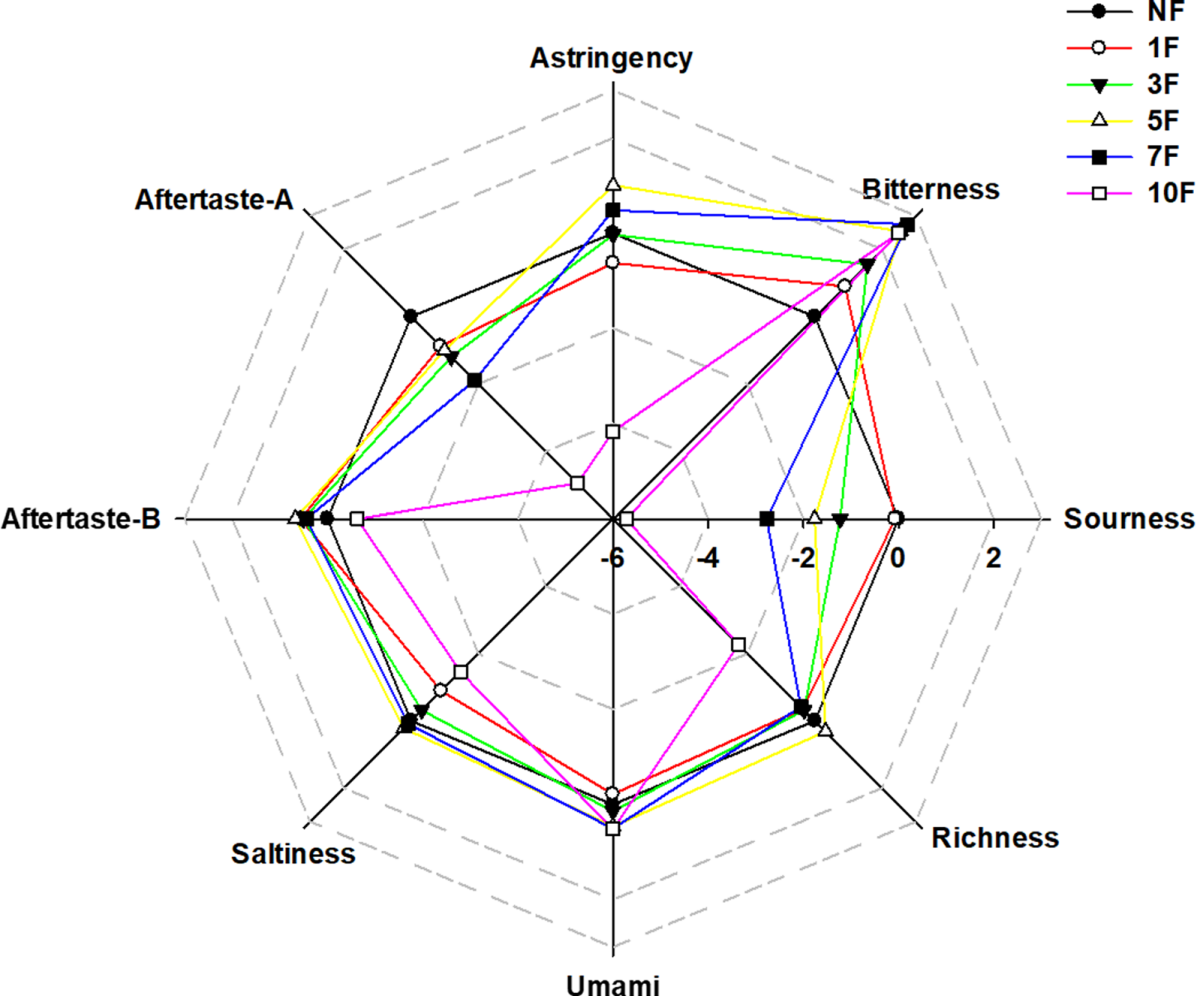
Radar map of taste sensory evaluation of fermented Yerba mate using *R. oligosporus* by electronic tongue sensor system

The results of taste sensory evaluation of unfermented and fermented Yerba mate are shown in Fig. 5. The sour taste decreased as the period of fermentation lengthened, with sourness values of − 0.07, − 1.23, − 1.76, − 2.77, and − 5.72 U for 1F, 3F, 5F, 7F, and 10F yerba mate, respectively, compared to unfermented yerba mate control. The bitter taste tended to increase as the period of fermentation increased, with bitterness values of 0.90, 1.56, 2.54, 2.74, and 2.48 U for 1F, 3F, 5F, 7F, and 10F yerba mate, respectively, compared to unfermented yerba mate control. Aftertaste-A, an aftertaste due to astringency, tended to decrease with longer fermentation, with values of − 0.87, − 1.19, − 0.99, − 1.88, and − 4.95 U for 1F, 3F, 5F, 7F, and 10F yerba mate, respectively, compared to unfermented yerba mate control. Samples with differences of > 1 U can be distinguished by a person (Kobayashi et al. 2010). The sour, bitter, and aftertaste-A tastes changed significantly from 3F. There were no significant differences between samples in umami or aftertaste-B, an aftertaste caused by acidic bitterness common in beer, coffee, etc. There were sudden decreases in astringency, saltiness, and richness only in 10F yerba mate, with values of − 4.17, − 1.46, and − 2.26 U, respectively. The samples fermented for shorter periods showed no significant differences in these taste values compared to the unfermented control.

## Discussion

SSF is a microbial fermentation process in which microorganisms are grown on insoluble materials in the near-absence or absence of free water. During SSF, the insoluble materials were used as the solid substrate, which acts as nutrients for the growth and metabolism of microorganisms along with the physical support for the fermentation process (Couto and Sanroman 2006; Pandey et al. 2000). Yerba mate contains 80.7% carbohydrates, 4.1% protein, 0.9% fats, and minerals and vitamins (Berté et al. 2011; Junior and Morand 2016). Therefore, it was used as a substrate in SSF with *R. oligosporus* to enhance its bioactive compounds, such as phenolics and saponins. Phenolic compounds and saponins are the key compounds that hand out the biological activities of Yerba mate. The total phenolic content of Yerba mate was risen to 20% by SSF using *R. oligosporus* (Fig. 2A). These TPC results were in agreement with our previous studies showing that SSF using *R. oligosporus* increased the TPC of wild-simulated ginseng leaves and wild turmeric (Lim et al. 2022, 2023). The saponins in Yerba mate are ursolic and oleanolic acid aglycons (Heck and De Mejia 2007), which contribute to its flavor and biological activities, including anti-inflammatory, hypocholesterolemic, and antiparasitic effects (Heck and De Mejia 2007). Although there was no significant difference in TSC among unfermented, 1F, and 10F Yerba mate, the saponin content of 7F Yerba mate was risen to 38% by SSF using *R. oligosporus* (Fig. 2B). Furthermore, the chlorogenic acid, caffeine, and caffeic acid in fermented Yerba mate were increased up to 5 day fermentation. Then, they were decreased by increasing the fermentation time to 7 day and 10 day. In contrast, ferulic acid and caffeine concentration increased when the fermentation time increased up to 10 day. Hur et al. (2018) reported that the chlorogenic acid content of quinoa increased to 1500% after 3F and then decreased to the control level after 5F using *R. oligosporus*. Another study showed that the amount of ferulic acid in wild turmeric rose by 28% after 7F using *R. oligosporus* (Lim et al. 2022). The increases in TPC, TSC, caffeic acid, ferulic acid, chlorogenic acid, and caffeine concentrations in Yerba mate could be explained by enzymes produced by *R. oligosporus*, like β-glucosidase, β-glucuronidase, xylanase, and protease, secreted by *R. oligosporus* that degrade cell wall structural components (Cerda et al. 2013; Nguyen Thai et al. 2014; Pinelo et al. 2006; Yadav et al. 2013) or conjugate saponins to release saponins and both free and bound phenolic compounds during fermentation (Correia et al. 2004; Lim et al. 2023; McCUE et al. 2003; Nguyen Thai et al. 2014; Vattem et al. 2004).

The antioxidant activities of fermented Yerba mate by *R. oligosporus* via FRAP and ORAC showed that ORAC values were increased by 92%, while FRAP values were raised to 15%. These results were similar to our previous studies in wild turmeric, buckwheat, quinoa, wild-simulated ginseng, and wild-simulated ginseng leaves (Hur et al. 2018; Lee et al. 2020; Lim et al. 2023, 2022; Park et al. 2017). The FRAP and ORAC values of fermented wild-simulated ginseng leaves and wild turmeric using *R. oligosporus* was increased up to 1.63- and 1.57-fold compared with unfermented controls, respectively (Lim et al. 2022, 2023). The antioxidant activities of wild-simulated ginseng were increased 3.72-fold by fermentation using *R. oligosporus* (Lee et al. 2020)*.* Intestinal α-glucosidases that hydrolyze carbohydrate to monosaccharides are membrane-bound enzymes found in the small intestinal epithelium. α-Glucosidase inhibitors provide secondary health benefits, like moderating plasma triglyceride levels and reducing the risk of cardiovascular disorders and hypertension (Benalla et al. 2010). The 1F Yerba mate extract expressed 1.06 times higher inhibitory activity against yeast α-glucosidase than unfermented Yerba mate extract. In contrast, the inhibitory effects of 7F and 10F Yerba mate were 1.06- and 1.18-fold lower than unfermented Yerba mate. Ranilla et al. (2010) demonstrated that Yerba mate water extracts could inhibit yeast α-glucosidase in a dose-dependent manner. Furthermore, Kang et al. (2012) reported that feeding Yerba mate to mice could reduce blood glucose levels. Yerba mate contains a dominantly of polyphenolic compounds such as chlorogenic acid, caffeine, caffeic acid, ferulic acid, rutin, and saponins that contribute to its antioxidant activities and antidiabetic via α-glucosidase and α-amylase inhibition (Colpo et al. 2016; Gülçin 2006; Miao and Xiang 2020; Oboh et al. 2015). Herein we found that the antioxidant activities and α-glucosidase inhibitory activity of fermented Yerba mate by SSF using *R. oligosporus* were increased when the total phenolic content, total saponin content, chlorogenic acid, caffeic acid, caffeine in Yerba mate were increased (Table 1, Figs. 2, 3). Together with decreasing the content of total phenolic, total saponin, chlorogenic acid, caffeic acid, and rutin, the antioxidant activities and α-glucosidase inhibitory activity of fermented Yerba mate was also decreased. Pearson’s correlation analysis between antioxidant activities and TPC of Yerba mate showed strong correlations with ORAC (*r* = 0.819) and FRAP (*r* = 0.974). Therefore, the increase in antioxidant activities and α-glucosidase inhibitory activity of fermented Yerba mate was ascribed to increased levels of phenolic and saponin contents.

By studying the effects of unfermented and fermented Yerba mate on the NO production by LPS-stimulated RAW264.7 cells, we found that the NO inhibitory effect of *R. oligosporus*-fermented Yerba mate at 1F was 1.35-fold higher compared to unfermented Yerba mate. A previous study reported that the anti-inflammatory effect of Yerba mate was mediated by the NF-κB pathway (Puangpraphant and De Mejia 2009). Yerba mate can suppress hepatic TNF-α and restore hepatic and muscle insulin signaling in mice with high-fat diet-induced obesity (Arçari et al. 2011). In another study, rising levels of the anti-inflammatory cytokine IL-10 and reduced levels of the proinflammatory cytokines TNF-α and IL-1β in adipose tissue were noted in rats treated with Yerba mate for 30 days (da Silva Lima et al. 2014). However, further studies are required to analyze the anti-inflammatory activity of fermented Yerba mate.

Chlorogenic and caffeic acids impart a sour and astringent taste (Chen et al. 2022). The dramatically decreased sourness and astringency of 10 day fermented Yerba mate can be due to the highly reduced chlorogenic and caffeic acid contents. Saponins have a bitter taste (Heng et al. 2006). Total saponin contents are the highest at 7 day fermented Yerba mate. It is highly correlated to the bitterness peak at the 7 day fermented Yerba mate. Aftertaste-astringency is positively related to polyphenols and flavonoids (Cheng et al. 2021). The drastic decrease in aftertaste-astringency came from the highly decreased contents of polyphenols such as chlorogenic acid, caffeic acid, and rutin at the 10 day fermented Yerba mate.

In conclusion, this study showed that SSF using *R. oligosporus* could significantly impact the biochemical characteristics and sensory evaluation of Yerba mate. The total saponin content, caffeic acid, and chlorogenic acid contents of Yerba mate were increased after 5 day fermentation and then reduced, while its TPC increased after 1 day fermentation and then decreased. The antioxidant activities of Yerba mate increased after 1 day fermentation but decreased with the prolongation of fermentation time. In addition, Yerba mate had higher anti-inflammatory and yeast α-glucosidase inhibitory activity after 1 day fermentation. Sensory evaluation showed that the taste of the 1 day fermented Yerba mate was similar to the control.

Overall, these results suggested that fermented Yerba mate could be utilized as a bioactive material in the pharmaceutical, food, and beverage industries. Furthermore, the results of this study contribute to our understanding of the advantages of SSF on Yerba mate and its potential uses in food, beverage, and pharmaceutical industries. However, the mechanism underlying the enhancing biological properties of fermented Yerba mate is required for further study.

### Supplementary Information

**Additional file 1: Table S1.** UPLC-PDA condition of standard sample. **Table S2.** LC-QDa mass conditions and summary of qualification results for linearity of standard compounds

## Data Availability

The data used to support the findings of this study are included with the article and supplementary files.
