# Supplementary material for: Phytochemical and functional characterization of fermented Yerba mate using Rhizopus oligosporus
Source: AMB Express. 2023 Sep 9;13:94. doi: 10.1186/s13568-023-01600-4 (PMC10492770; doi:10.1186/s13568-023-01600-4)
Supplement: Supplementary file 1 — Additional file 1: Table S1. UPLC-PDA condition of standard sample. Table S2. LC-QDa mass conditions and summary of qualification results for linearity of standard compounds [file 13568_2023_1600_MOESM1_ESM.docx]

**Additional file Materials**

**Phytochemical and functional characterization of fermented Yerba Mate using *Rhizopus oligosporus***

**So-Hyung Kwak** **^1^, Hayeong Kim^2^, Ji hyeon Jeon^3^, Kunal Pal^4^, Dong-Hyun Kang ^1, 5,^ *, Doman Kim** **^2, 3, 6,^ ***

^1^ Department of Agricultural Biotechnology, College of Agriculture and Life Sciences, Seoul National University, Seoul, 08826, Republic of Korea. Email: [shkwak16@snu.ac.kr](mailto:shkwak16@snu.ac.kr) (SH Kwak); [kang7820@snu.ac.kr](mailto:kang7820@snu.ac.kr) (DH Kang)

^2^ The Institute of Food Industrialization, Institutes of Green Bio Science &Technology, Seoul National University, Gangwon-do, 25354, Republic of Korea. Email: [hara2910@snu.ac.kr](mailto:hara2910@snu.ac.kr) (HY Kim); [kimdm@snu.ac.kr](mailto:kimdm@snu.ac.kr) (D Kim)

^3^Graduate School of International Agricultural Technology, Seoul National University, Gangwon-do, 25354, Republic of Korea. Email: [akreo96@snu.ac.kr](mailto:akreo96@snu.ac.kr) (JH Jeon)

^4^Department of Biotechnology and Medical Engineering, National Institute of Technology, Rourkela, 769008 India. Email: [kpal.nitrkl@gmail.com](mailto:kpal.nitrkl@gmail.com) (K Pal)

^5^Department of Food and Animal Biotechnology, Department of Agricultural Biotechnology, Center for Food and Bioconvergence, and Research Institute for Agricultural and Life Sciences, Seoul National University, Seoul, 08826, Republic of Korea

^6^Fervere Campus Corporation, Gangwon-do, 25354, Republic of Korea.

**Corresponding Author:**

**Doman Kim:** **E-mail**: [kimdm@snu.ac.kr](mailto:kimdm@snu.ac.kr); **Tel:** +82-33-339-5720; **Fax:** +82-33-339-571

**Dong-Hyun Kang:** **E-mail**: [kang7820@snu.ac.kr](mailto:kang7820@snu.ac.kr) **Tel:** +82-2-880-2697; **Fax:** +82-2-883-4928

**Table S1. UPLC-PDA condition of standard sample**

| **Compound** | **Concentration range**  **(μg/mL)** | **Absorbance**  **(nm)** | **Linearity**  **(R^2)^** | **Regression equation** |
| --- | --- | --- | --- | --- |
| Ergosterol | 0.1 – 60.0 | 280 | 0.999 | Y=46800X+5850 |

**Table S2.** LC-QDa mass conditions and summary of qualification results for linearity of standard compounds

| **Compound** | **Concentration range**  **(μg/mL)** | **M/Z** | **Polarity** | **Corn voltage**  **(V)** | **Capillary voltage**  **(kV)** | **Linearity**  **(R^2)^** | **Regression equation** |
| --- | --- | --- | --- | --- | --- | --- | --- |
| Chlorogenic acid | 0.1 – 10.0 | 355.0 | Positive | 5 | 1.3 | 0.98 | Y = 154323X - 49576 |
| Caffeic acid | 0.1 – 5.0 | 181.10 | Positive | 10 | 1.3 | 0.99 | Y = 299000X + 2300 |
| Ferulic acid | 0.1 – 5.0 | 195.13 | Positive | 10 | 1.3 | 0.99 | Y = 2960000X + 465000 |
| Caffeine | 0.1 – 5.0 | 195.17 | Positive | 10 | 1.3 | 0.98 | Y = 2960000X + 356000 |
| Rutin | 0.1 – 5.0 | 609.0 | Negative | 20 | 1.3 | 0.99 | Y = 165000X - 11500 |
